# Characterization and Comparative Profiling of MicroRNAs in a Sexual Dimorphism Insect, *Eupolyphaga sinensis* Walker

**DOI:** 10.1371/journal.pone.0059016

**Published:** 2013-04-19

**Authors:** Wei Wu, Qiuping Ren, Chengjun Li, Yanyun Wang, Ming Sang, Yi Zhang, Bin Li

**Affiliations:** Jiangsu Key Laboratory for Biodiversity and Biotechnology, College of Life Sciences, Nanjing Normal University, Nanjing, China; University of Nebraska Medical Center, United States of America

## Abstract

**Background:**

MicroRNAs are now recognized as key post-transcriptional regulators in animal ontogenesis and phenotypic diversity. *Eupolyphaga sinensis* Walker (Blattaria) is a sexually dimorphic insect, which is also an important source of material used in traditional Chinese medicine. The male *E. sinensis* have shorter lifecycles and go through fewer instars than the female. Furthermore, the males have forewings, while the females are totally wingless.

**Results:**

We used the Illumina/Solexa deep sequencing technology to sequence small RNA libraries prepared from the fourth-instar larvae of male and female *E. sinensis*. 19,097,799 raw reads were yielded in total: 7,817,445 reads from the female library and 11,280,354 from the male, respectively. As a result, we identified 168 known miRNAs belonging to 55 families as well as 204 novel miRNAs. Moreover, 45 miRNAs showed significantly different expression between the female and the male fourth-instar larvae, and we validated 10 of them by Stem-loop qRT-PCR. Some of these differentially expressed miRNAs are related to metamorphosis, development and phenotypic diversity.

**Conclusions/Significance:**

This is the first comprehensive description of miRNAs in *E. sinensis*. The results provide a useful resource for further in-depth study on molecular regulation and evolution of miRNAs. These findings not only enrich miRNAs for hemimetabolans but also lay the foundation for the study of post-transcriptional regulation on the phenomena of sexual dimorphism.

## Introduction

MicroRNAs (miRNAs) are tiny (∼22 nucleotides), endogenous, and non-coding RNAs that negatively regulate gene expression by binding to mRNA target sequences in eukaryotes [1]. MiRNAs have become a major class of post-transcriptional regulatory molecules, which play a key role in a wide diversity of biological processes such as development, metabolism and apoptosis in organisms [2], [3], [4], [5]. In addition, miRNAs have also been recognized as trustworthy data set to resolve phylogenetic problems in metazoan systematics due to the following properties: (1) new miRNA families are continually added to metazoan genomes; (2) mature sequence of miRNA accumulates mutations very slowly and (3) the rate of miRNA acquisition outweighs the rate of miRNA losses in most metazoan taxa [6]. Since the first member of miRNAs lin-4 was discovered in 1993 [7], miRNAs have been found in a variety of eukaryotic organisms ranging from sponges to mammals [8].

In recent years, next generation sequencing technology, such as solexa sequencing and 454 sequencing have become robust methods to identify numerous miRNAs in animals and plants [9], [10], [11], [12]. More than 21,264 miRNAs were deposited in the latest miRBase database release 19.0 in August 2012. Although insects account for a large propotion of known animals, so far, only 3457 miRNAs have been reported for insects and these miRNAs mainly restrict to Diptera (*Drosophila, Anopheles*. *gambiae*, *Aedes aegypti*, *Culex quinquefasciatus*), Hymenoptera (*Nasonia, Apis mellifera*), Coleoptera (*Tribolium castaneum*), Orthoptera (*Locusta migratoria*), Lepidoptera (*Bombyx morii, Heliconius melpomene, Manduca sexta*), and Homoptera (*Acyrthosiphon pisum)* (http://www.mirbase.org/).

The ground beetle (*Eupolyphaga sinensis* Walker) is a kind of insect used in traditional Chinese medicine. In folk medical practices, it could enhance immune response and promote blood circulation by removing blood stasis. In recent years, researchers have discovered antitumor and immunomodulatory effects of extracts from *E. sinensis* [13], [14]. Nevertheless, studies that have focused on the other biological characters of this species are scarce. *E. sinensis* is a species with sexual dimorphism. In general, the lifecycles of male ground beetles are shorter than the female. The male usually go through 7 instars while 9–11 instars in the female when they reach sexual maturity from their first instar larvae. Furthermore, phenotypic differences are also significant between both sexes. The male adults have forewings while females are totally wingless and have a bigger body size. Given that the scarcity of genetic information for *E. sinensis*, we thus largely focus on these differences in post-transcriptional level. Previous studies discovered a number of sex difference-related microRNAs from gonads of mammals [15], [16], [17], but these miRNAs were particularly reproduction-related. In the present study, we used Solexa high-throughput sequencing method to identify microRNAs for *E. sinensis* of both sexes. We totally obtained 160 known miRNAs, 8 known miRNA*s and 204 novel miRNAs. Among these, 45 miRNAs showed significantly different expression between the female and the male. The result will provide a better understanding of miRNA-mediated regulation in different sexes.

## Materials and Methods

### Sample preparation and RNA isolation

Two libraries for male and female larvae of *E. sinensis* were constructed in this study. Each 10 freshly ecdysed fourth instar larvae were pooled, as this stage is the earliest stage to distinguish their sex. All insects were reared in our lab. The total RNA was extracted from the whole bodies except the digestive tubes using the Total RNA Purification Kit (LC Sciences, Houston, USA), according to the manufacturer's protocol. Quantity and integrality of the total RNA was evaluated by Agilent2100 (Agilent, USA).

### Solexa sequencing

The sequencing procedure was previously described by Ma et al. [18]. In brief, the purified small RNAs (15–30 bases, bp) were ligated with a pair of adaptors (Illumina, San Diego, CA, USA) to their 5′ and 3′ ends. Then the small RNAs were amplified using the adapter primers for 17 cycles, and fragments of 70–90 bp (small RNA + adaptors) were excised from gels. The purified DNA fragments were used directly for cluster generation and sequencing analysis by the Genome Analyzer GAIIx (Illumina, San Diego, USA).

### Bioinformatic analysis of Solexa sequencing data

After masking of vector and adaptor sequences and removal of contaminated reads, the clean reads were then filtered for miRNA prediction with the proprietary ACGT101-miR v3.7 package (LC Sciences, Houston, USA). First, raw sequences that matched metazoan mRNA, rRNA, tRNA, snRNA, snoRNA, repeat sequences, and other ncRNAs deposited in the NCBI databases (http://www.ncbi.nlm.nih.gov/), RFam (http://rfam.janelia.org/), and Repbase (http://www.girinst.org/repbase/) were discarded. Then, we clustered the remaining reads based on sequence similarity and they were analyzed by BLAST against miRBase 19.0. Sequences in our libraries with identical or related (one mismatch) from metazoan mature miRNAs were identified as known miRNAs. The residual sequences after known miRNAs identification were aligned to the *T. castaneum* genome (ftp://ftp.bioinformatics.ksu.edu/pub/BeetleBase/3.0/) in order to identify potentially novel miRNAs. Certain target sequences around the small RNA were used to explore the secondary structure and folding energy (≤-15 kcal/mol) using Mfold software (http://mfold.rna.albany.edu/) and Vienna RNAfold (http://rna.tbi.univie.ac.at/cgi-bin/RNAfold.cgi).

### Verification of miRNAs by quantitative realtime PCR (qRT-PCR)

To validate relative expression levels of the identified miRNAs in our libraries, we selected 10 differentially expressed miRNAs for qRT-PCR. The total RNA was the same sample used in deep sequencing. The specific forward primers of 10 selected miRNAs were designed according to the sequence of miRNA itself, which were available in Table 1. The reverse transcription reaction was performed with the One Step PrimeScript RT reagent Kit (TaKaRa, Dalian, China) according to the manufacturer's protocol. The qRT-PCR was performed with SYBR Premix Ex Taq II (TaKaRa, Dalian, China) on the iCycler iQ real-time PCR detection system (Bio-Rad). Three biological replicates were performed for each sample and U6 snRNA was used as an internal reference. The relative expression level of miRNA was calculated according to the arithmetic formula, 2^−△△Ct^ [19].

**Table 1:** Primers used in this study for Quantitative real-time PCR.

Gene name	RT primer (5′–3′)	Forward primer (5′–3′)	Reverse primer (5′–3′)
KC-esi- miR-100	GTCGTATCCAGTGCAGGGTCCGAGGTATTCGCACTGGATACGACAACACA	GCGAACCCGTAGATCCGA	CAGTGCAGGGTCCGAGGTAT
esi-miR- 184b	GTCGTATCCAGTGCAGGGTCCGAGGTATTCGCACTGGATACGACTGCCCT	GGCTGGACGGAGAACTGA	CAGTGCAGGGTCCGAGGTAT
KC-esi- miR-375	GTCGTATCCAGTGCAGGGTCCGAGGTATTCGCACTGGATACGACTAACTC	GCGTTTGTTCGTTCGGCT	CAGTGCAGGGTCCGAGGTAT
esi-miR- 10-5p	GTCGTATCCAGTGCAGGGTCCGAGGTATTCGCACTGGATACGACTACAAA	GCGCTACCCTGTAGATCCG	CAGTGCAGGGTCCGAGGTAT
esi-miR- 125-5p	GTCGTATCCAGTGCAGGGTCCGAGGTATTCGCACTGGATACGACTCACAA	GGCGTCCCTGAGACCCTAA	CAGTGCAGGGTCCGAGGTAT
esi-miR-8	GTCGTATCCAGTGCAGGGTCCGAGGTATTCGCACTGGATACGACAGACAT	GGCGCGTAATACTGTCAGGTA	CAGTGCAGGGTCCGAGGTAT
esi-miR- 283-5p	GTCGTATCCAGTGCAGGGTCCGAGGTATTCGCACTGGATACGACCCAGAA	GCGGCAAATATCAGCTGGTA	CAGTGCAGGGTCCGAGGTAT
esi-miR- 12-5p	GTCGTATCCAGTGCAGGGTCCGAGGTATTCGCACTGGATACGACCCAGTA	GCGCGCTGAGTATTACATCAG	CAGTGCAGGGTCCGAGGTAT
esi-miR- 315	GTCGTATCCAGTGCAGGGTCCGAGGTATTCGCACTGGATACGACGGCTTT	GGGCGTTTTGATTGTTGCTC	CAGTGCAGGGTCCGAGGTAT
esi-miR- 3477-5p	GTCGTATCCAGTGCAGGGTCCGAGGTATTCGCACTGGATACGACTCACAG	GGCGGCTAATCTCATATGGTA	CAGTGCAGGGTCCGAGGTAT
U6	AACGCTTCACGATTTTGCG	GCTTCGGCGGTACATATAC	AACGCTTCACGATTTTGCG

## Results and Discussion

### Solexa sequencing of *E. sinensis* small RNAs

**Figure 1:**
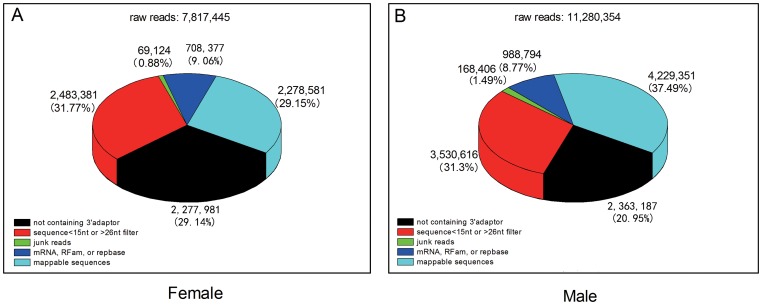
Statistics of small RNA sequences from the female (A) and male (B) *E. sinensis* libraries. The raw reads were classified into five separate categories, Mappable sequences states the raw reads were passed through a series of the digital filters by Illumina's Genome Analyzer Pipeline software and ACGT101-miR program, and used in the further miRNA identification.

**Figure 2:**
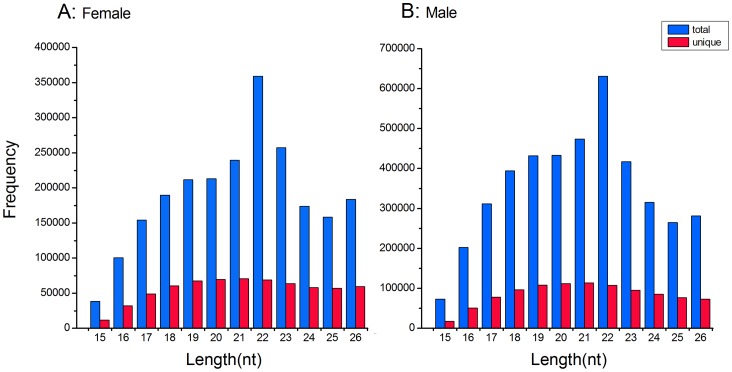
Size distribution of mappable reads related to miRNA in the female (A) and male (B) libraries of *E. sinensis*. The total represents the number of all mappable sequences were marked blue, and the unique represents the number of unique sequences were marked red. nt, nucleotides.

Two libraries of the small RNAs were constructed from their whole bodies except the digestive tubes of female and male fourth instar larvae of *E.sinensis*, and then the latest Solexa sequencing technology was used to identify miRNAs from these libraries. We obtained a dataset of 19,097,799 raw reads in total: 7,817,445 from the female and 11,280,354 from the male. After filtering sequences with low resolution, length less than 15 nts or larger than 26 nts, adapter sequences and junk sequences, 2,986,958 and 5,218,145 reads (with length from 15–26nt) were remained, respectively. We further discarded certain known types of RNA sequences, such as mRNA, rRNA, tRNA, snRNA, snoRNA and repetitive sequences as well. After these screening processes, 2,278,581 and 4,229,351 high-quality reads (Also called as: Mappable sequences) from the female and male samples were used for miRNA identification (Figure 1). Size distributions of the high-quality reads were similar in the female and male libraries (Figure 2). The peak was at the 22nt in each library, which was also observed in small RNA libraries of *B.germanica* [20], *L. migratoria* [21], *A. albopictus* and *C. quinquefasciatus* [22].

### Identification of known miRNAs from *E.sinensis*

The mappable sequences were aligned to all mature animal miRNA and miRNA precursor sequences in miRBase 19.0 (http://www.mirbase.org/). Among which, 102 miRNAs corresponding to known insect pre-miRNAs were mapped to the *T. castaneum* genome (Table S1), 58 miRNAs corresponding to other known metazoan pre-miRNAs in miRBase but could not be mapped to the *T. castaneum* reference genome, and they were labeled as KC (Known Candidate) (Table S2). Failure of mapping precursors of this large part of known microRNAs might be due to the lack of *E. sinensis* genome data or even transcriptome sequences. If the miRNA sequence with the most reads hit a known miRNA in miRBase, then the name of miRNA was used to represent this miRNA and the other variants. All the known miRNAs belongs to 55 different miRNA families. In addition, we also found several known but non-conserved miRNAs (miR-3049, miR-3770, miR-4944), which were only previously identified in one or two insect species.

**Figure 3:**
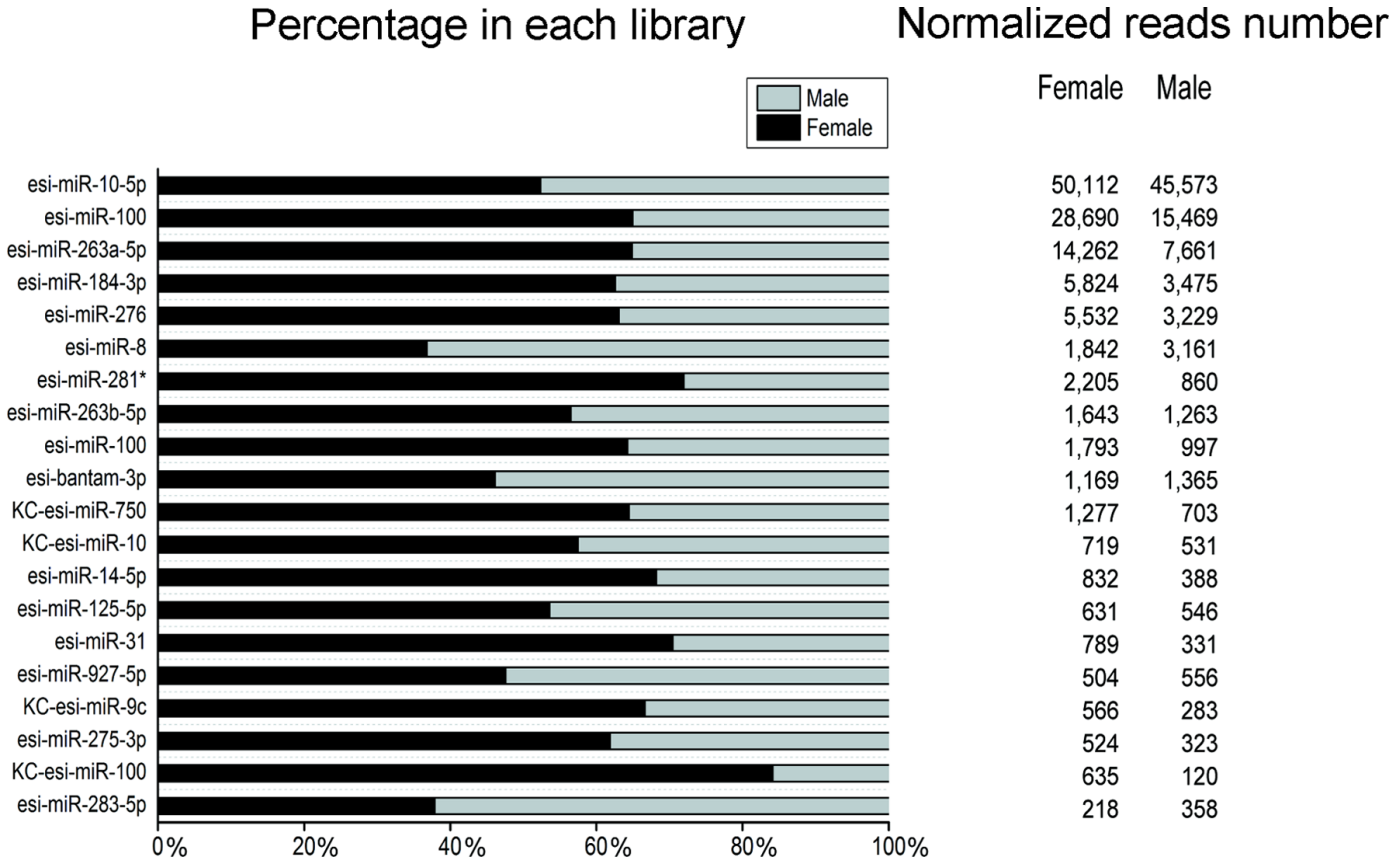
The most 20 abundant miRNAs identified in *E. sinensis*. The left panel was Relative expression levels between female (black) and male (light gray). The right panel was the normalized reads number.

In our dataset, a large proportion of known miRNAs were ubiquitously expressed (>10 reads) in both libraries. The most abundant miRNA was miR-10-5p with a total of over 300,000 reads, followed by miR-100, miR-263a-5p, miR-184-3p and miR-276 (Figure 3; Table S3). These miRNAs were also highly abundant in other insects,such as miR-1, miR-275, miR-276 and miR-8 in *L. migratoria* [21]; miR-1, let-7, miR-31a, miR-275 and miR-276a in *Blattella germanica* [20]. Concerning the functions of these abundant miRNAs in our libraries, miR-10 is highly conserved in sequence and genomic position. It is mainly involved in modulating the Hox gene function in insects [23]. MiR-100 and miR-276 are closely related to metamorphosis and they are preferentially expressed in specific instar larvae in *B. mori* and *D. melanogaster* [24], [25]. In *Drosophila*,miR-184, strongly expressed in the germline, regulates several distinct steps during oogenesis and early embryogenesis [26].

### Identification of known miRNA* from *E.sinensis*

In most cases, miRNA*s are undetectable by conventional methods because miRNA*s will be degraded soon after being exported to cytosol. During the process of identifying known miRNAs, 8 miRNAs that matched to the same precursor sequences with their mismatched complementary mature miRNAs were also detected, which might be miRNA* sequences (miR-8*, miR-9a*, miR-133*, miR-190*, miR-276*, miR-281*, miR-932*, miR-993a*). In fact, these 8 miRNA*s only accounted for a small portion of known miRNAs. On the whole, the abundance of miRNA*s was much lower than that of their corresponding miRNAs except for miR-281*, which was over 40 times higher. This kind of phenomenon was consistent with the instability of miRNA* during biogenesis. Various miRNA* sequences with high amounts have also been identified in other libraries by deep sequencing [20], [21], [27], [28]. This phenomenon may result from the evolutionary process called “arm switching”, which was previously mentioned in fly, red flour beetle [29] and worms [30]. The molecular mechanisms of arm switching are not fully under­stood and deserve further discovery. Meanwhile, we should not ignore the fact that some unexpected miR*/miR ratios can be influenced by adapter and library preparation of high-throughput sequencing [31]. The functions of miRNA*s have been previously ignored because they were usually regarded as important primarily for maintaining the miRNA precursor secondary structure [32]. Nowadays, researchers have gradually focused on their important roles in regulating gene expression [32]. Unfortunately, functional studies of miRNA*s have been carried out mostly in vertebrates but not in insects.

### Identification of novel miRNAs in *E.sinensis*

After obtaining the above-mentioned known miRNAs, the remaining sequences were aligned to the *T. castaneum* genome to forecast potentially novel miRNAs, since the *E. sinensis* genome has not been sequenced yet. Based on the criteria that the extended sequences at the aligned genome locations have the propensity of forming hairpin structures, a total of 204 novel miRNAs were predicted (Table S4). The negative folding free energy (dG in kcal/mol) of these predicted pre-miRNAs varied from −15 to −52.1 kcal/mol. In order to verify the existence of the predicted miRNAs, we used the reverse transcription polymerase chain reaction (RT-PCR) for 10 randomly chosen novel miRNAs. As a result, these 10 miRNAs were all amplified out (data not shown), demonstrating that deep sequencing is a valid method to discover novel miRNAs from the non-model species. The number of reads for these predicted novel miRNAs varied from 1 to 149, much less than that for conserved miRNAs. Previous studies indicated that non-conserved miRNAs were often expressed with an organ- or developmental-specific pattern [33], [34], [35], [36], [37], [38], [39]. We actually just identified a part of novel miRNAs in *E. sinensis* due to the fact that both RNA libraries were only constructed from fourth instar larvae. The low abundance of novel miRNAs might suggest a specific role for these miRNAs under various circumstances, in specific organs, or during certain developmental stages. It remains to be investigated whether these low-abundant miRNAs are expressed at higher levels in other developmental stages or regulated by environmental stress.

### Variations of mature miRNAs

It is recognized that a majority of miRNAs are highly evolutionarily conserved throughout different animal taxa, however, sequence variations including single nucleotide polymorphism (SNP) and length difference still existed. Then the sequences of known miRNAs were analyzed in our libraries and they were divided into several categories (Table S5): (1) 5′ ends variations; (2) variations next to the “seed regions” and (3) 3′ ends variations [40]. The ‘seed region’ of microRNAs (2–8 bases) binds to those target mRNAs by perfect complementarity in most of the microRNA-target interactions. Thus, it is so conserved that any base substitution occurring at ‘seed region’ may lead to the change of targets. In our analyses, uridines and adenosines (U+A = 81.5%) are the preferred bases at the first position of 5′ end (Table S1, S2), which are ubiquitous in the miRNA world. Base addition and base deletion at the beginning of 5′ end were thought to have profound affects in the past, because of a seed shifting would directly interfere with targets recognition and further result in the variation of phenotypic characteristics [41]. In the following research, evidence has arisen that base addition at 5′ end does not always change the seed sequence [42]. Base substitutions frequently appear at a region next to the ‘seed region’, namely 9–15 nt. That most changes occur at the 3′ end of mature sequence is the result of variation in Drosha or Dicer processing and non-templated 3′ nucleotide addition [43], which leads to diversification of miRNAs. The 3′ end sequence of a mature miRNA is believed to be a minor contributor in determining miRNA target specificity. Nevertheless, it may alter stability of miRNA:mRNA binding, thus it is crucial for determining the strength of its binding with target mRNA [44]. It is believed that sequence variations are likely to be one of the driving powers in the evolution of miRNAs.

### Differential expression of miRNAs between two libraries

**Figure 4:**
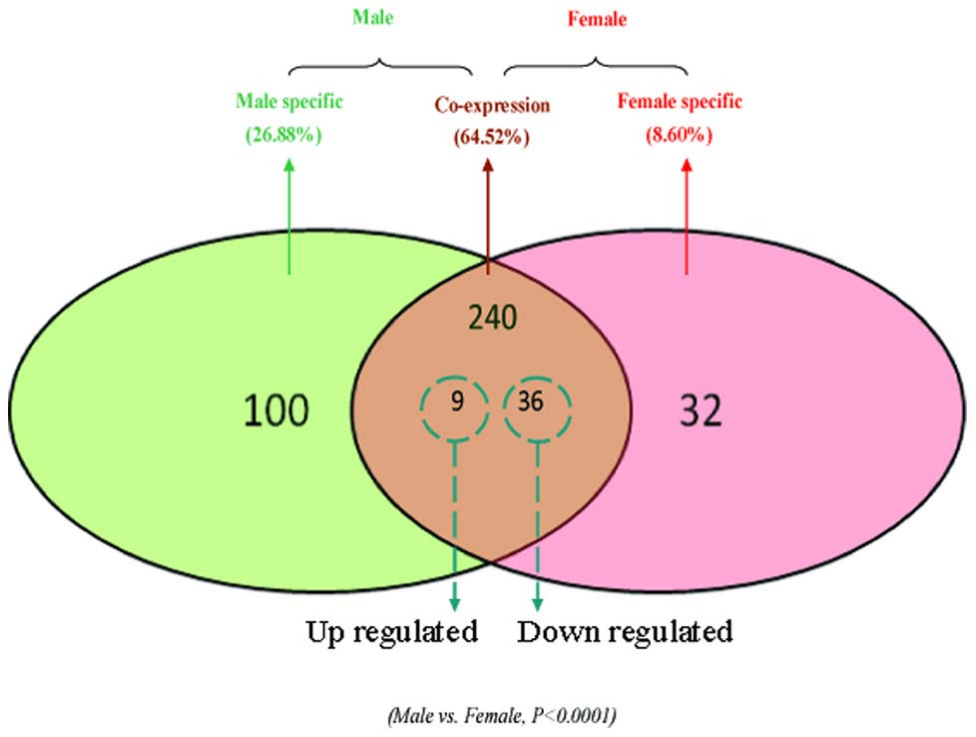
Comparison of miRNAs between the female and male *E. sinensis*. The Venn diagram displays the distribution of 372 unique miRNAs between the male (left, green circle) and female (right, pink circle) libraries. The dashed circles indicate the miRNAs that were significantly differentially expressed (p<0.0001, Bonferroni corrected) in the two samples.

**Figure 5:**
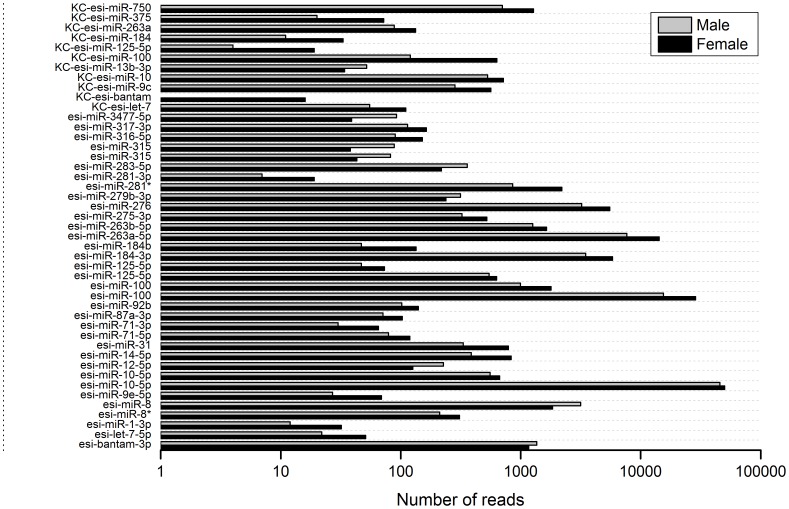
Comparison of the differentially co-expressed miRNAs between the female and male *E. sinensis*. 45 miRNAs showed significant differential expression between the female (black) and male (light gray) libraries (p-value <0.0001). The frequency of miRNAs in two libraries were normalized.

**Figure 6:**
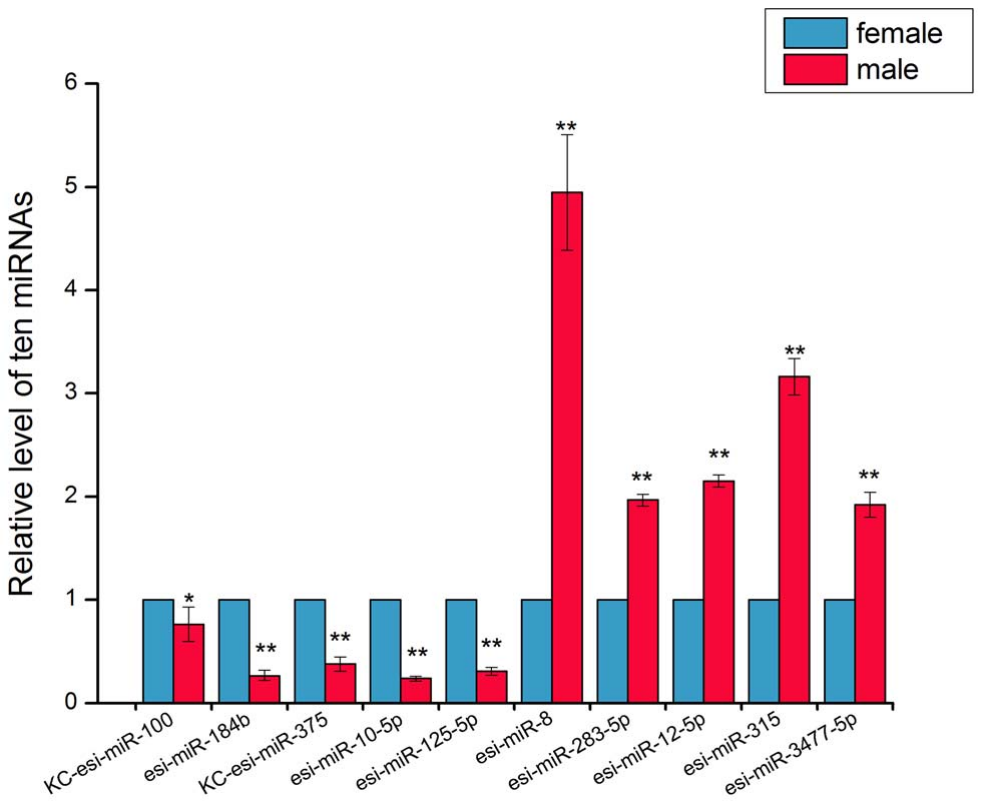
Relative expression levels of ten in the female and male *E. sinensis* by Quantitative real-time PCR. “*” and “**” means a statistically significant difference at level p<0.05 and p<0.001, respectively, for this miRNA in the female and male *E. sinensis*. The error-bars show standard deviation for three biological replicates.

We found that the other unknown small RNAs accounted for a large portion of the mappable reads in both female and male libraries (Table 2), which indicated that miRNAs were only a small part of the small RNAs world. In this study, we totally identified 372 miRNAs from the male and female libraries. Among these, 100 miRNAs (26.88%) were male-specific, 32 miRNAs (8.60%) female-specific and other 240 miRNAs (64.52%) were co-expressed (Figure 4). Given the fact that the male library consisted of additional 3,462,909 raw reads, it is acceptable that we have discovered much more specific miRNAs in the male library than that in the female. Furthermore, we can not ignore that almost all the gender-specific miRNAs are with less than 10 reads or even 1 read. It is hard for us to verify whether these miRNAs are truly gender-specific or just the result of coverage difference for libraries. To determine the significance of the observed differences in reads for those co-expressed miRNAs, we utilized the IDEG6 program (http://telethon.bio.unipd.it/bioinfo/ IDEG6_form/), which performs a normalization calculation to adjust for the two libraries. The expression levels of miRNAs in the two libraries were normalized using the following formula: Normalized expression  =  (Actual miRNA sequencing reads count/Total mappable reads count) ×1,000,000. Results of the Audic and Claverie test, Fisher exact test, and Chi-squared 2×2 test with a Bonferroni correction for multiple comparisons and a p value <0.0001 indicated that differences in the miRNA reads were statistically significant. As a result, 45 miRNAs showed significant differential expression between the female and male libraries (Figure 5). Among these, 36 miRNAs (80%) were down-regulated while 9 ( 20%) were up-regulated (male/female) (Table S6). The miRNA expression level in a library was usually measured by the reads percentage in the library. Stem-loop qRT-PCR was thought to be an efficient method to confirm the expression pattern of differentially expressed miRNAs [19]. We then carried out qRT-PCR for 10 miRNAs and the selection criterion was to cover a wide spectrum of reads number, because previous study indicated Solexa sequencing data with less than 100 reads could only roughly represent their relative abundance or serve as reference [20]. All 10 miRNAs showed similar expression patterns as those revealed by our Solexa sequencing (Figure 6), which further validated high-throughput sequencing indeed a robust method to identify and quantify miRNAs.

**Table 2:** Annotations of mappable reads in the female and male libraries of *E. sinensis*.

	Female	Percentage(%)	Male	Percentage(%)
conserved miRNA candidates	276160	12.12%	379727	8.98%
known miRNA candidates	8715	0.38%	8632	0.20%
specific miRNA candidates	523	0.02%	1265	0.03%
unkown small RNAs	1993183	87.48%	3839727	90.79%
mappable reads	2278581	100%	4229351	100%

In recent years, crucial biological functions of miRNAs have been discovered in insects but mostly limited to holometabolous insects, such as *D. melanogaster*. Nowadays, we know that miRNAs regulate important biological processes such as apoptosis, cell division, Notch signaling, metamorphosis, neural development and oogenesis in the fly. Differential expression levels of miRNAs in different life stages of a certain insect may suggest their possible roles in regulation of metamorphosis and development. The genome of *E. sinensis* has not been sequenced yet, which is an obstacle for further studying miRNAs of *E. sinensis* and their targets. To our knowledge, many miRNAs are not only evolutionary conserved in sequence but also function within insecta, which lay a solid foundation for our research.

*E. sinensis* is a kind of hemimetabolous insect whose metamorphosis process is not so complicated as holometabola, but various differentially expressed miRNAs are metamorphosis-related or development-related, which are well studied in *Drosophila*.

The let-7 family is phylogenetically conserved in diverse bilaterians with a conserved function in regulation of heterochronic development [45]. In *Drosophila,* mir-100, 125 and let-7 gene cluster are co-regulated. The expression levels of these 3 genes are regulated by juvenile hormones and ecdysone, which control moulting and metamorphosis processes in insects [46]. Interestingly, miR-100, miR-125 and let-7 in the male *E. sinensis* are all down regulated, which demonstrate a similar co-regulated effect in *E. sinensis*. In *B. mori*, the miR-9c is normally expressed at the egg and pupae stages but it has the highest level in larvae and a much lower level in pupae. This dramatic alternation of expression indicates that miR-9c may regulate larvae to pupae metamorphosis [47]. Moreover, another two miRNAs miR-275 and miR-276 are metamorphosis-related. MiR-275 is up-regulated from the third instar larvae to pupae and down-regulated during pupal metamorphosis of both sexes, while miR-276 is preferentially expressed in feeding and spinning larvae [24],[35]. In our results, miR-9c, miR-275 and miR-276 are all down regulated in the male *E. sinensis*, which indicate that the male and the female are under different stages of maturity/metamorphosis. Importantly, the male *E. sinensis* have shorter life cycles than the female. Larvae of the male usually go through 7 instars until they reach sexual maturity, while 9–11 instars are common for larvae of the female. Even, we chose the fourth instar larvae for both sexes during samples preparation. Nevertheless, maturity/metamorphosis stage of the male larvae should precede the female larvae. Consequently, different expression levels of these miRNAs are in accordance with their phenomena on the maturity/metamorphosis.

*E. sinensis* is a sexual dimorphism insect and the morphological differences are significant characteristics to distinguish the female from the male. For example, female individuals are generally with bigger body sizes, especially in adults. The adult male individuals have forewings while the adult female are totally wingless. In *Drosophila*, miR-14 is identified as a cell death suppressor. Animals lacking miR-14 are usually stress sensitive and have reduced lifespans [48]. Intriguingly, the expression level of miR-14 in female sample is significantly higher than that in male sample. This result is consistent with the body size difference between genders. On the other hand, the differentially expressed level of miR-14 also reveals a reduced lifespan of the male *E. sinensis*, which is an indisputable fact. Bantam, targeting the *Mei-p26* gene, mainly controlled the growth rate of normal tissue by regulating cell growth, cell division and apoptosis in *Drosophila*, and the Bantam mutant individuals were smaller than the wild type [49],[50]. As a result, a candidate miRNA, KC-esi-bantam is 16 times abundant in female than in male. Although the reads for this miRNA are low in both samples, we still have reason to believe that this miRNA is related to the sexual dimorphism of *E. sinensis* to some extent. Wg/Wnt signaling is now well studied and highly regulated in insecta, regulating the development of the wing. Abnormal activation or inhibition of Wg/Wnt pathway has resulted in developmental defects and diseases in silkworm [35]. MiR-315 is a potent activator of wingless signaling in fruit fly [51] and moreover it is thought to play an important function in the wing development along with other two miRNAs: miR-8 and miR-9a. It is worth noting that miR-315 and miR-8 are both up-regulated in the male sample. Both female and male larvae of the fourth instar are wingless, but the male will have forewings during the metamorphosis from larva to adult. Thus, the higher expression levels of miR-315 and miR-8 in the male should play potential roles in wing formation and development.

In fact, researche on insect miRNAs has lagged somewhat behind that of mammals, nematodes and plants. Thus, the functions of manydifferentially expressed miRNAs in this study remain unclear, and are also not well studied in *Drosophila*. To further explore the functions of these miRNAs, comprehensive target prediction and functional assays should be carried out. To date, there has been no 3′UTR database or complete genomic database for *E. sinensis* or any species of Blattaria. Thus, it seems to be difficult for us to predict targets of *E. sinensis* with currently available information. However, in this study, we at least reveal that these miRNAs are closely related to the differences of metamorphosis, development and phenotype between female and male *E. sinensis*. This study no doubt expands the repertoire of insect miRNAs and will make contribution to further studies on post-transcriptional regulation on the phenomena of sexual dimorphism.

## Supporting Information

Table S1 Sequences and number of reads for known miRNAs and miRNA*s found in ***E. sinensis.***
(XLS)

Table S2 **Sequences and number of reads for known candidate miRNAs found in** ***E. sinensis.***
(XLS)

Table S3 **The most 20 abundant miRNAs identified in** ***E. sinensis*** **.**
(XLS)

Table S4 **Sequences and number of reads for novel miRNAs predicted in** ***E. sinensis.***
(XLS)

Table S5 **Variation pattern and alignment of mature miRNA sequences among different species.**
(XLSX)

Table S6 **Comparison of the differentially co-expressed miRNAs between the female and male** ***E. sinensis.***
(XLS)
